# Cellular Dynamics of Transgenic Porcine Endothelial Cells to Inflammatory Stimuli in Xenotransplantation Settings

**DOI:** 10.1111/xen.70149

**Published:** 2026-06-28

**Authors:** Mitra Gultom, Nina Thomi, Kassandra Teixeira‐Riberio, Jane Shaw, Alain Despont, Nikolai Klymiuk, Elisabeth Kemter, Eckhard Wolf, Robert Rieben

**Affiliations:** ^1^ Department for Biomedical Research University of Bern Bern Switzerland; ^2^ Klinik Und Poliklinik für Kardiologie Großtiermodelle Für Die Kardiovaskuläre Forschung TUM Klinikum Klinikum Rechts Der Isar Munich Germany; ^3^ Chair for Molecular Animal Breeding and Biotechnology Gene Center and Department of Veterinary Sciences LMU Munich Munich Germany

**Keywords:** coagulation, complement, endothelium, inflammatory responses, xenotransplantation

## Abstract

Inflammatory responses have been shown to contribute significantly to the rejection of grafts in both allo‐ and xenotransplantation. In particular, they play a pivotal role in promoting endothelial activation, complement deposition, and thrombosis, thereby compromising the graft function. In this study, we investigated the molecular and functional properties of genetically modified porcine aortic endothelial cells (PAECs) that carry a knockout of α1,3‐galactosyltransferase and express human CD46 and thrombomodulin (3GM) in xenogeneic and inflammatory environments. Transcriptomic profiling revealed that these genetic modifications effectively reduced the intrinsic inflammatory and procoagulant phenotype of 3GM PAECs. Under xenogeneic activation, 3GM PAECs also exhibited minimal cellular responses distinct from those of wild‐type (WT) PAECs, along with robust protection from the activation of the complement and coagulation systems. However, under inflammatory conditions, 3GM and WT PAECs showed more aligned transcriptional and functional profiles characterized by pronounced upregulation of proinflammatory and prothrombotic pathways, increased complement deposition, and a shift toward a more procoagulant state. This loss of protection during inflammatory conditions was associated with the induction of inflammatory and procoagulant mediators, including PAI‐1 and uPAR, despite stable transgene expression. Collectively, these insights enhance our understanding of the complex interplay between inflammation, complement, coagulation, and immune regulation in xenotransplantation. Our findings further emphasize the importance of incorporating both genetic and pharmacologic strategies targeting inflammatory pathways to enhance graft compatibility.

## 1 Introduction

While allotransplantation remains the clinical standard for patients with end‐stage heart failure, the disparity between organ supply and demand continues to drive interest in alternative approaches, such as xenotransplantation. The emergence of genetically modified pigs has revolutionized this field, enhancing the compatibility of pig organs with the human immune system. The triple genetically modified (3GM) pigs, featuring the knockout of α1,3‐galactosyltransferase (*GGTA1*) to eliminate the major xenoantigen and the expression of human complement regulatory protein CD46 (hCD46) as well as human thrombomodulin (hTBM), are among the most extensively studied genetic modification combinations [1, 2, 3]. With these relatively minimal genetic alterations, preclinical studies using 3GM pig hearts in baboons have demonstrated encouraging results, including prolonged graft survival and functional support, indicating a reasonable degree of xenograft immune acceptance [2, 3]. More recently, even more complex genetic modifications, such as the 10GM pigs, have been developed and tested not only in non‐human primates but also in decedent models and two compassionate‐use cases in humans [4, 5, 6].

While those preclinical and clinical achievements represent significant milestones in the xenotransplantation field, they also underscore the complexity of immune barriers that currently remain unresolved and need to be overcome to achieve long‐term survival of pig organs in humans. In preclinical and human studies, dysregulation of the complement and coagulation cascades, as well as thrombotic microangiopathy, remain a formidable challenge in achieving long‐term xenograft survival [4, 5, 7]. This highlights the need for a deeper investigation into the cellular and molecular mechanisms that drive the activation of the plasma cascade systems mediated by innate immune rejection of xenografts.

Inflammatory responses following transplantation play a crucial role in the acute and delayed rejection in both allogeneic and xenogeneic transplant settings [8, 9, 10, 11]. The release of inflammatory factors such as IL‐6 and TNF‐α has been shown to impair graft survival [12, 13]. Endothelial cells (ECs), the primary point of contact between the xenograft and the recipient's circulation, play a crucial role in mediating both inflammatory and coagulative responses [14, 15]. Moreover, xenoantigen deletion and transgene expression in genetically modified pigs initially exert their protective functions on the endothelial surface as they interact with the recipient's blood, highlighting the critical role of the endothelial layer in determining graft fate.

The role of EC protection and the impact of genetic modifications have been previously explored in vitro [13, 16, 17, 18]. However, the contribution of inflammatory stimuli on a cellular level in the context of xenotransplantation remains insufficiently investigated. Moreover, since endothelial phenotype and function are strongly influenced by hemodynamic forces, incorporating dynamic shear stress and flow conditions that reflect in vivo physiology into in vitro models is essential [19, 20]. In this study, we address this gap by characterizing the molecular and cellular responses of genetically modified porcine ECs under defined flow conditions and simulating both xenogeneic and inflammatory environments. By combining transcriptome analysis with functional assays assessing complement and coagulation protection, we aimed to dissect the cellular responses and pathways involved in the loss of protection triggered by inflammatory stimuli in xenotransplantation. Ultimately, such insights provide a critical fundamental knowledge to overcome the complex immunological and thrombotic challenges in xenotransplantation.

## 2 Materials and Methods

### 2.1 Primary Porcine Endothelial Cells

Primary porcine aortic endothelial cells (PAECs) were isolated from the thoracic aorta, which is a byproduct of licensed experimental work, obtained from euthanized female and male Landrace pigs aged 4–8 months. 3GM pigs were homozygous for alpha‐1,3‐galactosyltransferase knockout (GGTA1‐KO) and heterozygous for human CD46 (hCD46) and hTBM (generated by the Institute of Molecular Animal Breeding and Biotechnology, LMU Munich, Germany and Revivicor, Blacksburg, USA). For cell isolation, the cells were removed mechanically from the lumen of the aorta using a humidified cotton swab and cultured on a fibronectin‐coated disk. Complete media, consisting of DMEM (Gibco, 10566016) supplemented with 10% fetal bovine serum (FBS), 100 IU penicillin, and 100 µg/mL streptomycin, 1% endothelial growth medium 2 supplement mix (PromoCell C‐39216), was used for EC culturing. Cells were grown in a humidified incubator (37°C, 5% CO_2_). During cell expansion, fibroblast cells were mechanically removed from the EC colony. Upon confluence, cells were phenotyped using a goat polyclonal antibody against porcine CD31 (R&D Systems MAB33871), VE‐Cadherin (RnD systems, AF938), and α‐smooth muscle actin (α‐SMA, Abcam, ab7817) to visualize the endothelial markers and to check for purity using immunofluorescence microscopy (Figure S1). To confirm the genetic modification, cells were stained for α‐Gal presence (lectin from *Griffonia simplicifolia* [Sigma, L‐2140]), hCD46 (Hycult Biotech, HM2103), and hTBM (Abcam, ab6980) (Figure S2). Cells were cryopreserved, and for experiments, cells up to passage six were used to minimize the risk of endothelial phenotype shifting. ECs obtained from two different donor animals for each 3GM and wild‐type (WT) were used.

### 2.2 Culturing and Treatment of PAECs in a Microfluidic System

To culture the PAECs in a microfluidic system, 100,000 cells in 100 µL of complete media were seeded in a μ‐slide VI 0.4 (Ibidi, 80606), as previously described [16, 21]. Before seeding, the μ‐slide was coated with 100 µL of 10 µg/mL fibronectin (Merck, FC010) for 30 min at 37°C. Cells were incubated overnight before being connected to a peristaltic pump (Gilson, Minipuls 3) using sterile silicone tubings to introduce the flow. Media consisting of DMEM supplemented with 10% FBS, 1% glutamine, 1% BSA (Sigma, A7030), and 4% dextran (Sigma, 31390) was used as the flow medium. The laminar shear stress was adjusted to 10 dyn/cm^2^ and maintained for 72 h in a humidified incubator at 37°C and with 5% CO_2_. The media was refreshed every day.

For simulating xenogeneic activation, cells were perfused with 3 mL of flow media containing 10% pooled normal human serum (NHS) obtained from three healthy donors for 2 h. To simulate the inflammatory conditions and the activated endothelium state due to systemic inflammation in xenotransplantation, cells were primed with 100 ng/mL recombinant human TNF‐α (RnD Systems, 210‐TA‐020) for 4 h before NHS perfusion, as previously described in prior studies [16, 20]. To test IL‐6 influence on complement deposition, cells were treated with 1 µg/mL IL‐6 (RnD Systems, 206‐IL‐010) for 4 h before NHS perfusion, which concentrations were selected based on similar cell activation to the working concentration of TNF‐α (Figure S3a). Thereafter, cells were fixed and followed by immunofluorescence analysis.

### 2.3 Immunofluorescence Analysis

For immunofluorescence staining, cells were fixed with 4% formaldehyde solution for 15 min at room temperature (RT), followed by a blocking step at RT with PBS containing 3% BSA. Next, cells were incubated with primary antibody diluted in the antibody solution (1% BSA, 0.05% Tween 20 in PBS) for 2 h at RT, or overnight at 4°C. A mouse antibody against E‐selectin (Sigma, S9555) was used to check cell adhesion marker expression. To visualize antibody deposition, goat anti‐human IgG (Sigma, F5512) and IgM (Sigma, F5384) were used. Rabbit antibody against C3b/c (DAKO, F0201) and mouse antibody against C5b‐9 (Thermo Fisher, DIA‐011‐01‐02) were used to visualize complement deposition. Subsequently, cells were incubated with secondary antibody labeled with fluorophores diluted in the antibody solution: donkey anti‐goat IgG (H + L) conjugated with Alexa Fluor 633 (Invitrogen, A21082), donkey anti‐mouse IgG (H + L) conjugated with Alexa Fluor 488 (Invitrogen, A32766), and donkey anti‐rabbit IgG (H+L) conjugated with Alexa Fluor 568 (Invitrogen, A10042). DAPI was included in the secondary antibody mix to visualize nuclei. Imaging was performed using a 20 × objective on a Zeiss LSM 980 confocal microscope. Figures were analyzed using ImageJ (version 2.14.0/1‐54f) and assembled using the FigureJ package [22, 23]. Brightness and contrast were adjusted identically to the controls. Quantification of the immunofluorescence signal was performed by measuring the area above threshold on three randomly acquired images for each channel.

### 2.4 Coagulation Assay

A coagulation assay was performed by perfusing the cells in the microfluidic channel with recalcified citrated human plasma. Before perfusion, the cells were washed with calcium‐ and magnesium‐free PBS 3x and subsequently perfused with human citrated plasma spiked with 25 mM CaCl_2_ immediately before perfusion. Shear stress was maintained at 10 dyn/cm^2^. The coagulation time was determined when complete occlusion of the channel occurred.

### 2.5 Bulk RNA Sequencing and Data Analysis

Total cellular RNA from the PAECs was isolated using the NucleoSpin RNA kit (Macherey‐Nagel, 740955) according to the manufacturer's guidelines, followed by quantification of the total RNA with a Quantifluor RNA system (Promega, E3310). Bulk RNA barcoding and sequencing (BRB‐seq) was performed by Alithea Genomics, as described previously [24]. A total of 200 ng of cellular RNA from four independent biological replicates was used to generate BRB‐seq libraries, which were then sequenced on an Illumina HiSeq 4000 platform. Five million raw reads per sample were generated. Demultiplexing, QC, alignment, and count matrix generation were performed using the BRB‐seq pipeline.

For gene expression analysis, the raw counts were normalized and log1P transformed using the DESeq2 package (version 4.3.0). Differential gene expression analysis was performed with DESeq2 with an adjusted *p* value cutoff of 0.05 and an absolute log2FC cutoff of 0.58. Visualizations of overlapping DEGs amongst samples were performed using Venndiagramm package (Version 1.7.3). Pathway enrichment analysis was performed using the clusterProfiler package (version 4.12.6) with a false discovery rate cutoff of 0.1. Further data analysis and visualization were performed using a variety of additional packages in R (Version 2025.05.1 +513).

### 2.6 RT‐qPCR

cDNA was synthesized from total RNA using the High‐Capacity cDNA Reverse Transcriptase Kit (Thermo Fisher, 4374967c) according to the manufacturer's protocol. Two microliters of diluted cDNA were amplified with SYBR Green PCR Master Mix (Thermo Fisher, 4385612) according to the manufacturer's protocol, using specific primers targeting human and porcine thrombomodulin (TBM), and human CD46 (Table 1). Primers were designed and validated to be species‐specific, with no cross‐amplification between species genes at 35 amplification cycles. GAPDH was used as the reference gene. Measurements and analysis were performed with the Quantstudio 3 and associated software (Applied Biosystems). Relative gene expression was calculated using the 2^ΔΔCt^ method. Data are shown as fold induction of treated samples compared to those of untreated controls.

**TABLE 1:** Primer list for qPCR measurement of human and porcine CD46 and TBM.

Gene	Human	Porcine
CD46	F: GAT CGG AAT CAT ACA TGG CTA CC	F: GTGATGAGCCACCGAAGTTTG
	R: GCT TGG CCA TTT AAA GGA TCC C	R: GACGGAAGAGGTGGGAAGC
TBM	F: TCC CAG ATC GGC TCG CTG	F: GTCTGTCCCTATGAAATGGTCCTC
	R: GTG GGC ACG GCT CGA C	R: CTTCTCCCCAACACCCCAG
GAPDH	F: GAAGGTGAAGGTCGGAGTCAAC	F: CGATGGTGAAGGTCGGAGTG
	R: CAGAGTTAAAAGCAGCCCTGGT	R: TGCCGTGGGTGGAATCATAC

### 2.7 Statistical Data Analysis

All data are presented as the mean ± standard deviation (SD). The statistical analysis was performed using GraphPad Prism 10 (version 10.4.1). For statistical analysis, a *t* test and multiple comparisons using ordinary one‐way ANOVA, followed by the Fisher LSD test, were performed. All experiments were independently replicated at least three times.

## 3 Results

### 3.1 3GM Porcine Endothelial Cells Exhibit Reduced Proinflammatory and Procoagulant Phenotypes

To investigate the cellular responses of 3GM PAECs, cells were isolated from the aortic arteries of genetically modified pigs and cultured under physiological shear stress (10 dyn/cm^2^) for 72 h using Ibidi μ‐slides. To mimic xenogeneic activation in xenotransplantation, PAECs were perfused with media containing 10% pooled NHS. Inflammatory conditions were simulated by pre‐treating PAECs with recombinant human TNF‐α for 4 h before NHS perfusion. To characterize transcriptomic changes, we performed bulk RNA barcoding and sequencing (BRB‐seq) on 3GM and WT PAECs.

**FIGURE 1:**
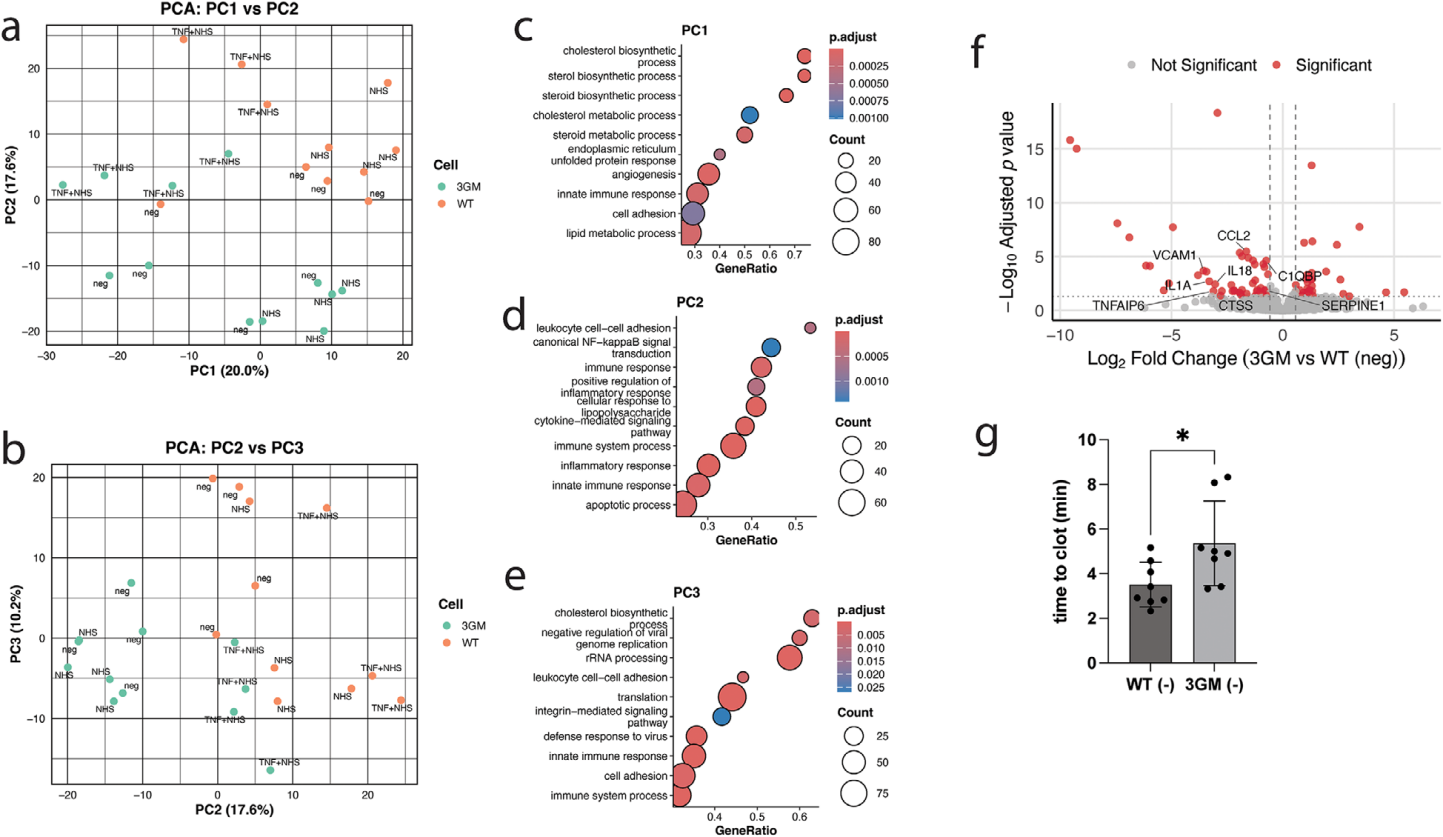
Transcriptomic signature of 3GM compared to wild‐type (WT) porcine aortic endothelial cells (PAECs). (a, b) Principal component analysis (PCA) of bulk RNA‐seq data from 3GM and WT PAECs cultured under flow (10 dyn/cm^2^ for 72 h) that are either cultured with standard media (neg), treated with normal human serum (NHS), and combination of recombinant human TNF‐α and NHS (TNF+NHS). (c–e) Gene set enrichment analysis of principle components (PCs) 1–3 loadings. (f) Volcano plot of differentially expressed genes (DEGs) between untreated 3GM and WT PAECs. (g) Time to clot formation in untreated 3GM and WT PAECs when perfused with recalcified citrated plasma. An unpaired *t* test was used for statistical analysis. Asterisks indicate statistically significant differences (* *p* < 0.05).

Principal component analysis (PCA) of the RNA‐seq data from all treatments revealed segregation between 3GM cells and WT PAECs, especially untreated and NHS‐treated 3GM PAECs, with the most prominent separation occurring along principal component (PC) 2 and 3 (Figure 1a, b, Figure S4). While gene loadings contributing to PC1 were mainly enriched for metabolic processes, PC2 and PC3 were substantially enriched for immune‐related pathways, including processes associated with innate immune recognition, cytokine responses, and inflammation (Figure 1c–e). These findings indicate that immune response pathways represent predominant transcriptomic alterations resulting from genetic modifications introduced into 3GM donor pigs for xenotransplantation.

Differential gene expression analysis between untreated 3GM and WT PAECs identified downregulation of several genes involved in innate immunity and inflammation, such as *VCAM1*, *CCl2*, *IL18*, *IL1A*, and *TNFAIP6*, which are commonly associated with leukocyte recruitment, cytokine signaling, and inflammatory activation (Figure 1f, Table S1). *CTSS* (cathepsin S, involved in MHC Class II processing) downregulation was also observed, suggesting possible alterations in antigen processing and adaptive immune responses. Notably, several genes involved in complement and coagulation pathways, including *SERPINE1* (*PAI‐1*) and *C1QBP* (C1q binding protein), were downregulated in 3GM PAECs. This transcriptome alteration corresponds with functional changes, as coagulation assays showed significantly prolonged clotting times on the surface of 3GM PAECs compared to WT PAECs when perfused with recalcified citrated human plasma (Figure 1g). These results indicate that the genetic modifications implemented in 3GM pigs result in reduced basal proinflammatory and procoagulant properties of the endothelial surface.

### 3.2 Cellular Responses of 3GM Endothelial Cells Under Xenogeneic and Inflammatory Conditions

**FIGURE 2:**
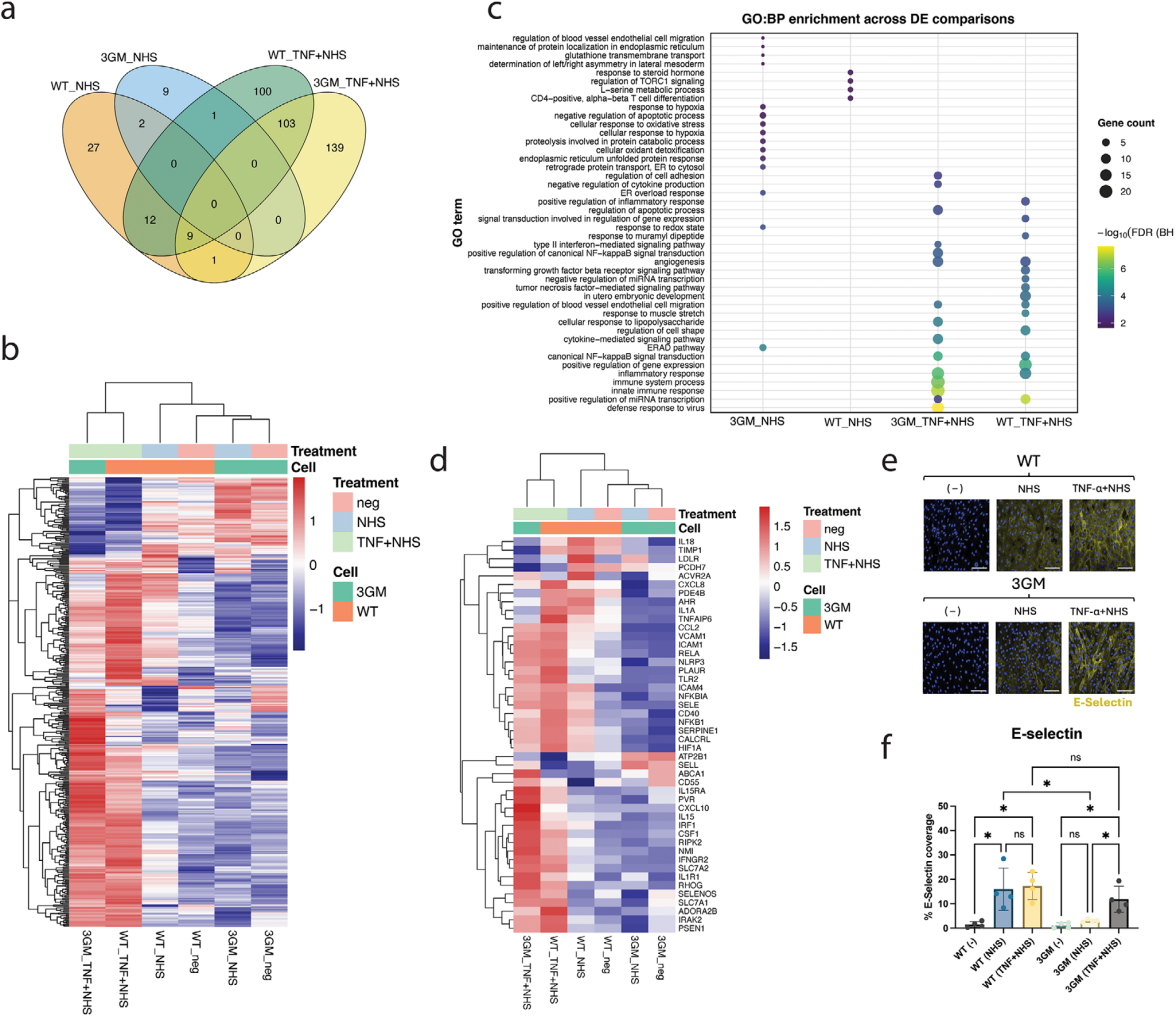
Genetically modified porcine aortic endothelial cells (3GM PAECs) exhibit minimal transcriptomic responses to human serum but respond more similarly to wild‐type (WT) PAECs under inflammatory conditions. (a) Venn diagram showing the number of differentially expressed genes (DEGs) identified in WT and 3GM PAECs under normal human serum (NHS) or TNF+NHS treatment compared to untreated controls. (b) Hierarchical clustering heatmap of all DEGs across conditions. The expression level is shown as the log‐transformed average normalized counts from four independent replicates. (c) Gene Ontology (GO) enrichment analysis of DEGs in both WT and 3GM PAECs following NHS or TNF+NHS stimulation. (d) Expression heatmap of key inflammatory response genes. (e) Immunofluorescence staining for E‐selectin (SELE) protein expression after NHS alone or after TNF‐α + NHS treatments (f). Quantification of E‐selectin expression from four replicates. Statistical analysis was done using one‐way ANOVA with multiple comparisons. Asterisks indicate statistically significant differences (* *p* < 0.05). Scale bar = 50 µm.

To assess the cellular responses to xenogeneic and inflammatory stimuli, we analyzed global transcriptional changes in 3GM and WT PAECs following exposure to NHS or a combination of TNF‐α + NHS, relative to untreated controls. NHS treatment alone elicited a minimal transcriptomic response in 3GM PAECs, with only 12 DEGs identified; only 2 DEGs were shared with WT PAECs under the same condition (Figure 2a, Table S1). In contrast, WT PAECs exhibited a broader transcriptional response, with 51 DEGs identified. Meanwhile, TNF‐α priming before NHS treatment resulted in 252 DEGs in 3GM PAECs, with a significant overlap with WT PAECs (Figure 2a). Hierarchical clustering of all DEGs revealed that while NHS treatment alone had a minimal impact on 3GM EC transcriptomes, TNF+NHS treatment resulted in expression patterns more aligned with those of WT PAECs (Figure 2b).

Pathway enrichment analysis further highlighted these differences (Figure 2c). In WT PAECs, NHS treatment induced enrichment of pathways that were notably absent in 3GM PAECs. However, under inflammatory conditions, both cell types shared enrichment of pathways associated with inflammatory responses, NF‐KB signaling transduction, EC migration, and angiogenesis (Figure 2c). A closer examination of the genes associated with inflammatory responses, including adhesion molecules (*VCAM1*, *ICAM1*, and *SELE*) and cytokine signaling mediators (*CSF1*, *CXCL8*, *CXCL10*, *CCL2*, *IL15RA*, and *IL1R1*), revealed that only TNF+NHS‐treated 3GM PAECs exhibited more aligned expression profiles with those of WT cells (Figure 2d). In contrast, some of these genes were already upregulated in WT cells when treated with NHS alone.

To validate these findings at the protein level, we assessed the expression of one of the DEGs, E‐selectin (SELE), which is a key adhesion molecule involved in inflammation. Immunofluorescence analysis and quantification confirmed minimal E‐selectin expression in 3GM PAECs after NHS treatment, while robust upregulation comparable to WT PAECs was observed following TNF‐α stimulation, consistent with transcriptome data (Figure 2e, f). In addition, we also show that TNF‐α alone induced a similar level of inflammatory cell phenotype in both WT and 3GM cells, as shown by similar level of E‐selectin expression after TNF‐α treatment (Figure S3b, c).

### 3.3 Complement and Coagulation Responses of 3GM PAECs Under Xenogeneic and Inflammatory Conditions

**FIGURE 3:**
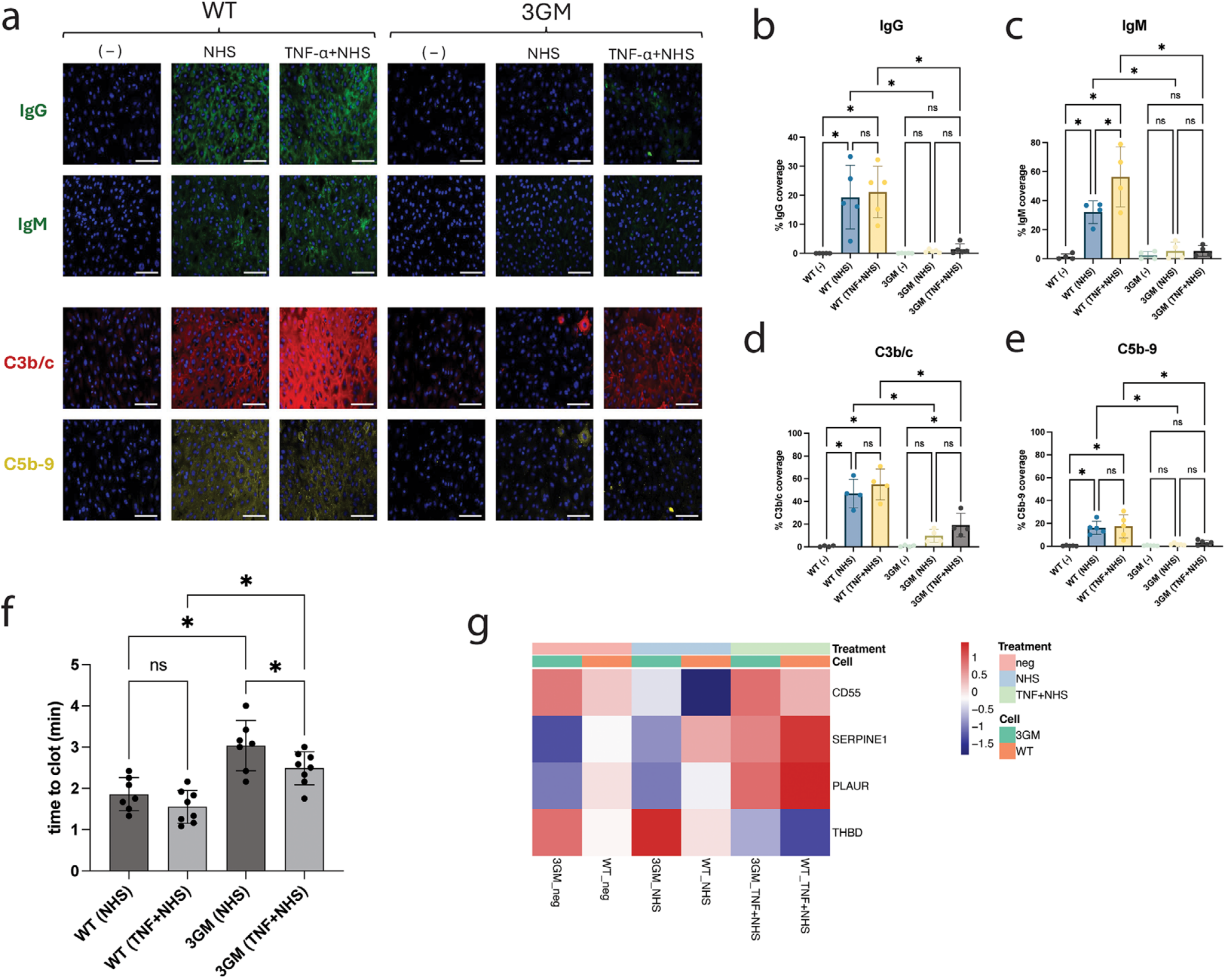
Inflammatory conditions compromise complement and coagulation protection in genetically modified (3GM) endothelial cells. (a) Immunofluorescence analysis of the deposition of human IgG, IgM, C3b/c, and C5b‐9 on wild‐type (WT) and 3GM porcine aortic endothelial cells (PAECs) after treatment with normal human serum (NHS) or TNF+NHS. Scale bar = 50 µm. Quantification of the coverage of (b) IgG, (c) IgM, (d) C3b/c, and (e) C5b‐9. Statistical analysis was done using one‐way ANOVA with multiple comparisons. (f) Clotting time assays using recalcified citrated human plasma were performed on 3GM and WT PAECs under both NHS and TNF+NHS conditions. (g) Transcriptomic profiles of complement and coagulation‐related genes in WT and 3GM PAECs. Asterisks indicate statistically significant differences (* *p* < 0.05).

Given that complement and coagulation activation are central to xenograft rejection in xenotransplantation, we next assessed the extent of complement deposition and coagulation activation on 3GM PAECs under xenogeneic and inflammatory stimuli. We observed that under NHS exposure, 3GM PAECs exhibited minimal IgG and IgM deposition, as well as the deposition of complement components (C3b/c and C5b‐9), indicating an effective reduction of complement activation (Figure 3a–e). However, upon treatment with TNF+NHS, we observed a significant increase in the deposition of C3b/c on 3GM PAECs, despite no increase in the deposition of IgG and IgM (Figure 3a–d). This suggests that inflammatory activation increases the complement activation on 3GM PAECs, potentially through alternative or lectin pathway mechanisms, rather than classical antibody‐dependent routes.

To assess the coagulation state, we also performed clotting assays using recalcified citrated human plasma on the PAECs after treatment with NHS and TNF+NHS. 3GM PAECs exhibited significantly longer clotting times than those of WT PAECs on both NHS and TNF+NHS‐treated conditions (Figure 3f). Nevertheless, we observed a significant reduction in clotting time in 3GM PAECs when treated with TNF+NHS compared to NHS alone, confirming the increased procoagulant state in the 3GM PAECs (Figure 3f). Moreover, at the transcriptomic level, we observed significant downregulation of porcine TBM (*THBD*) and upregulation of *SERPINE1* and *PLAUR* in 3GM cells under TNF+NHS treatment, but not under NHS treatment, corroborating the functional observations and possibly explaining the loss of coagulation protection. We confirmed that the expression of the human transgenes TBM and CD46 remained unchanged with either treatment (Figure S5).

**FIGURE 4:**
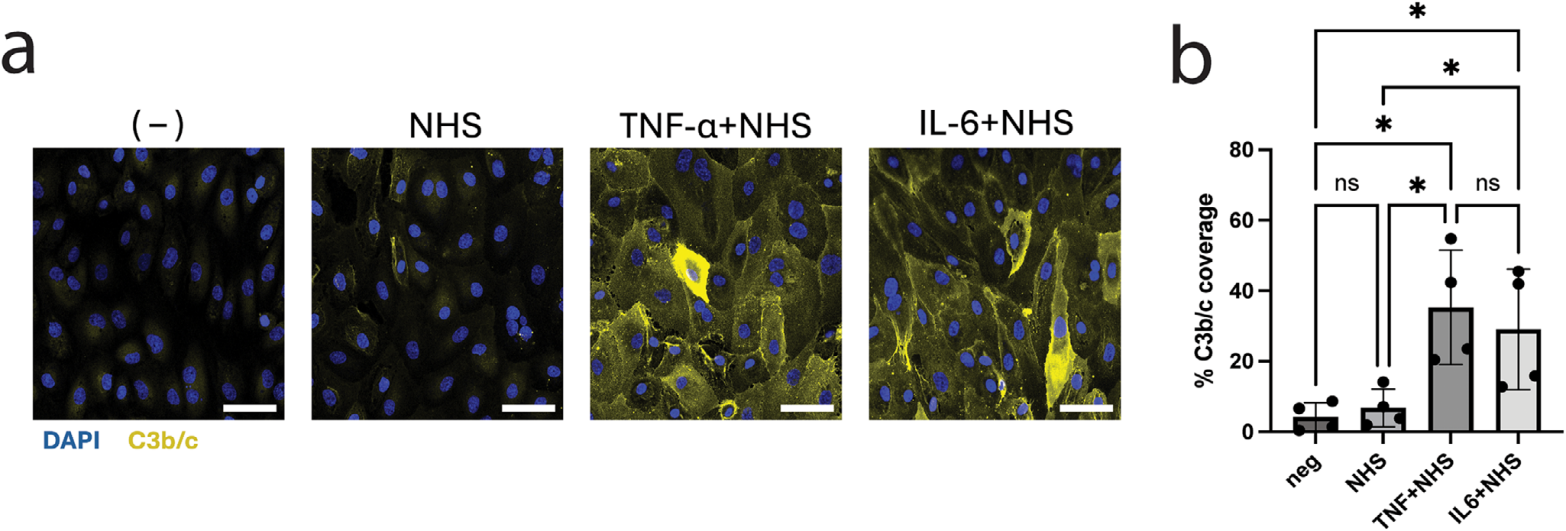
Inflammatory conditions triggered by human IL‐6 also increased complement activation in genetically modified (3GM) endothelial cells (ECs). (a) Immunofluorescence analysis of the deposition of human C3b/c on 3GM ECs after treatment with normal human serum (NHS), TNF‐α +NHS, or IL‐6+NHS. Scale bar = 50 µm. (b) Quantification of the coverage of C3b/c deposition on 3GM ECs. Statistical analysis was done using one‐way ANOVA with multiple comparisons. Asterisks indicate statistically significant differences (* *p* < 0.05).

To further evaluate whether inflammatory mediators other than TNF‐α can similarly compromise endothelial protection, we next examined the effect of IL‐6, as this cytokine has been widely implicated in systemic inflammation in xenotransplantation [25, 26]. Following pretreatment with IL‐6 in the presence of NHS, 3GM PAECs exhibited a significant increase in C3b/c deposition, comparable to that observed with TNF‐α plus NHS treatment (Figure 4a, b). These results further confirmed that inflammatory stress is a relevant driver of complement activation on 3GM PAECs. Taken together, these findings demonstrate that although 3GM PAECs are effectively protected against complement and coagulation activation under xenogeneic conditions, this protective phenotype is compromised under inflammatory stress.

## 4 Discussion

Inflammatory responses, both systemic and localized within the graft, are known to play a crucial role in xenograft rejection during xenotransplantation [8, 10]. In this study, we investigated the cellular responses of genetically modified 3GM PAECs to inflammatory stimuli, with a focus on their effects on complement activation and coagulation pathways. We showed that while these cells maintained adequate protection under xenogeneic conditions, inflammatory stress induced significant transcriptomic changes as well as loss of complement and coagulation protection. These findings underscore the critical influence of inflammatory microenvironments in compromising graft survival.

We show that genetic modifications in the 3GM endothelium are associated with reduced inflammatory responses and enhanced anticoagulant and profibrinolytic properties, marked by the downregulation of genes involved in immune cell interactions, cytokine‐mediated signaling, and responses to pathogens. This suggests a significant alteration of the basal anti‐inflammatory endothelial phenotype. The observed effects may be attributed to the expression of human CD46 and TBM, both of which are known to modulate inflammation and cytokine responses [27, 28, 29]. However, reduced inflammatory responses may also lead to increased susceptibility to specific pathogens as well as a less efficient link to the induction of adaptive immune responses, as recently highlighted in previous studies [30]. Further characterization of the influence of genetic modification on the pigs for xenotransplantation beyond the context of xenogeneic interaction is therefore necessary.

While we have previously described the complement and endothelial glycocalyx dynamics in 3GM cells during inflammatory conditions, our current study extends these findings by characterizing the transcriptomic responses of 3GM PAECs under xenogeneic and inflammatory conditions [16]. Under xenogeneic stimulation alone, 3GM PAECs exhibited minimal transcriptomic alterations. They showed minimal activation of key pathways of immune system processes, indicating a robust tolerance to xenogeneic stress. However, under inflammatory conditions, 3GM PAECs demonstrated a transcriptomic shift toward a proinflammatory phenotype, closely resembling that of WT PAECs. More importantly, we showed that the inflammatory state also correlated with increased complement deposition and a procoagulant profile in 3GM PAECs. These findings further confirm that while genetic modifications in 3GM PAECs offer protection under xenogeneic conditions, this protection may be compromised in the presence of inflammation, potentially contributing to xenograft rejection.

Systemic inflammatory effects have been shown to play a major role in xenotransplantation, even in the presence of an immunosuppressive regime, as shown by increased circulating IL‐6 and CRP, and MCP‐1 [10, 26]. Although IL‐6 and CRP are key systemic inflammatory markers in xenotransplantation, we did not observe significant upregulation of IL‐6 or CRP in pig ECs, likely because the graft ECs are not the primary source of these mediators. This suggests that elevated circulating IL‐6 and CRP reported in vivo likely reflect recipient‐derived systemic inflammation. In contrast, inflammatory conditions induced significant upregulation of multiple pro‐inflammatory mediators and chemoattractants in 3GM PAECs, including MCP‐1 (CCL2). These endothelial‐derived signals are consistent with previous in vivo reports and indicate that EC activation may promote innate immune cell recruitment and amplification of inflammation and a positive feedback loop, thereby contributing to sustained inflammatory responses despite immunosuppression [26, 31]. While our in vitro model does not fully capture the complexity of in vivo xenotransplantation, these data support a role for endothelial inflammatory activation under inflammatory conditions.

Our findings demonstrate that the complement deposition on 3GM PAECs during inflammatory conditions was increased without significantly elevated antibody binding, suggesting that increased complement activation could occur via mechanisms other than the classical pathway. A possible mechanism is activation through the alternative pathway, which is induced by proinflammatory cytokines such as TNF‐α [32]. The alternative pathway has been implicated in the early loss of xenografts due to uncontrolled complement activation and is a known contributor to ischemia‐reperfusion injury, a common complication in both allogeneic and xenogeneic transplantation settings [33, 34]. Further investigation is warranted to elucidate the precise molecular mechanisms by which inflammatory stimuli modulate complement activation on porcine xenografts. Understanding these pathways is critical for the development of more targeted strategies to mitigate complement‐mediated injury in xenotransplantation.

Our findings confirm that the observed loss of complement and coagulation regulatory function in 3GM PAECs under inflammatory conditions is not due to changes in the expression levels of the transgenes, that is, hCD46 and hTBM. Transcriptomic analysis also revealed a significant upregulation of key coagulation‐associated factors, including SERPINE1 (PAI‐1) and PLAUR (uPAR), in response to inflammatory stimuli, but not under xenogeneic stimulation alone. This suggests that a prothrombotic shift in 3GM cells during inflammatory stress is likely due to an impaired fibrinolytic balance. The increased expression of PAI‐1, the primary regulator of uPA and tPA activity, could lead to suppressed plasmin generation and reduced fibrin degradation, thereby promoting a hypercoagulable state [35, 36]. Concurrently, the upregulation of uPAR during inflammation has been described, along with increased soluble uPAR, which is associated with a poor prognosis in transplantation [37, 38, 39]. These findings underscore the need for further investigation into the molecular interactions between porcine‐derived coagulation modulators such as porcine PAI‐1 and uPA, and human blood components. In addition, our data suggest that the PAI‐1/uPA axis may play an essential role in the inflammatory procoagulant response of xenografts and could serve as a target to restore hemostatic balance and improve graft compatibility in xenotransplantation settings.

Our findings further emphasize the importance of regulating inflammatory responses in xenotransplantation. Persistent or excessive inflammation not only contributes to endothelial activation and dysregulation of coagulation but may also undermine the efficacy of existing genetic modifications. To address this, the integration of anti‐inflammatory genes, such as A20 and heme oxygenase‐1 (HO‐1), has shown promise in preclinical models and may be essential for achieving long‐term graft survival [40, 41]. Beyond genetic approaches, the use of existing or the development of novel anti‐inflammatory therapeutics tailored for xenotransplantation, such as cytokine and TNF‐α inhibitors, might be necessary [42, 43]. However, in view of the evidence from early human xenotransplantation experience, that complement activation persists despite current genetic modifications, additional context‐dependent strategies targeting inflammation‐driven complement and coagulation activation may be necessary to reduce xenograft injury and improve graft survival. EC protective molecules, such as low‐molecular‐weight dextran sulfate and multimeric sulfated tyrosine, which can maintain the endothelium in a quiescent state, may have great potential as an adjunctive treatment in xenotransplantation [44, 45]. Such interventions may prove particularly valuable during critical phases to enhance graft protection and functional integration.

In summary, our study provides detailed insights into the molecular mechanisms underlying inflammation‐associated loss of complement and coagulation regulation in genetically modified pig grafts used in xenotransplantation. We showed that upregulation of cellular inflammatory responses, as well as procoagulant and antifibrinolytic factors observed under inflammatory stress, likely contributes to the development of a prothrombotic and inflammatory phenotype. Therefore, refined strategies, such as anti‐inflammatory genetic modifications and tailored pharmacological interventions, may be essential for achieving sustained graft survival and functional compatibility in future xenotransplantation applications.

While this study focuses on inflammatory stress induced by TNF‐α, further detailed characterization using additional cytokines known to mediate inflammation in xenotransplantation, specifically IL‐6, would be necessary. In addition, other inflammatory conditions such as combinations of diverse stimuli, sera derived from recipients of rejected xenografts, as well as potential anti‐inflammatory therapeutics, can further shed light on the role of inflammatory responses in xenotransplantation. Moreover, inclusion of additional genetic backgrounds (such as GGTA1 KO alone, individual transgenes, or alternative combinations) as well as human ECs in future studies will be valuable. As such, these approaches can further delineate the specific contribution of each genetic modification to endothelial inflammatory, complement, and coagulation responses. Such comparative analyses would also improve the translational relevance of these findings to the human clinical xenotransplantation setting.

## Author Contributions

M.G. was involved in the conceptualization, investigation, methodology, and manuscript writing. N.T., K.T.‐R., A.D., and J.S. were responsible for the investigation and methodology. E.W., N.K., and E.K. generated the genetically modified pigs, participated in cell procurement, reviewed the manuscript, and were involved in the funding acquisition. R.R. was responsible for funding acquisition, conceptualization, investigation, and reviewing the manuscript.

## Conflicts of Interest

Authors Eckhard Wolf and Elisabeth Kemter are cofounders of the company XTransplant GmbH, Starnberg. The remaining authors declare that they have no conflicts of interest.

## Supporting information




**Supporting Information Figure S1**: Endothelial phenotype characterization. Representative images of WT and 3GM cells stained for endothelial cell markers CD31 (green), VE‐Cadherin (Red). α‐SMA (yellow) staining confirms the absence of fibroblasts and phenotypic shift, with porcine vascular smooth muscle cells (VSMCs) as a positive control. Scale bar = 50 µm.

**Supporting Information Figure S2**: Confirmation of the genetic modification in 3GM porcine endothelial cells. Representative images of WT and 3GM PAECs stained with *Griffonia simplicifolia* isolectin B to visualize the presence of α‐Gal, and species‐specific antibodies to visualize human thrombomodulin (hTBM) and human CD46. Scale bar = 50 µm.

**Supporting Information Figure S3**: Effects of inflammatory stimuli on endothelial cell (EC) activation. (**a**) Dose‐dependent effects of TNF‐α (1–100 ng/mL) and IL‐6 (10 ng/mL–1 µg/mL) stimulation, measured by increased E‐selectin expression. (**b**) E‐selectin expression in WT and 3GM cells following 4 h stimulation with 100 ng/mL TNF‐α. (**c**) Image‐based quantification of E‐selectin expression from three independent replicates. Asterisks indicate statistically significant differences (*p* < 0.05). Scale bar = 50 µm.

**Supporting Information Figure S4**: Scree plot from RNA‐seq principal component analysis (PCA) of all cells and treatments, displaying the variance explained by each principal component using 20 gene loadings.

**Supporting Information Figure S5**: Transgene and endogenous expression of CD46 and thrombomodulin. (**a**) Human CD46 and (**b**) human thrombomodulin expression in 3GM PAECs. (**c**) Porcine thrombomodulin expression in both WT and 3GM PAECs. Expression is displayed as the fold induction of expression in NHS or TNF+NHS‐treated cells compared to untreated cells. Statistical analysis was done using one‐way ANOVA with multiple comparisons. Asterisks indicate statistically significant differences (* *p* < 0.05).

**Supporting Information Table S1**: Lists of identified DEGs.

## Data Availability

Transcriptome data have been deposited in the ArrayExpress open‐access public repository with accession number E‐MTAB‐15491 and will be made publicly available upon publication.
