# Supplementary figures and images for: Cellular Dynamics of Transgenic Porcine Endothelial Cells to Inflammatory Stimuli in Xenotransplantation Settings
Source: Xenotransplantation. 2026 Jun 28;33(4):e70149. doi: 10.1111/xen.70149 (PMC13310968; doi:10.1111/xen.70149)

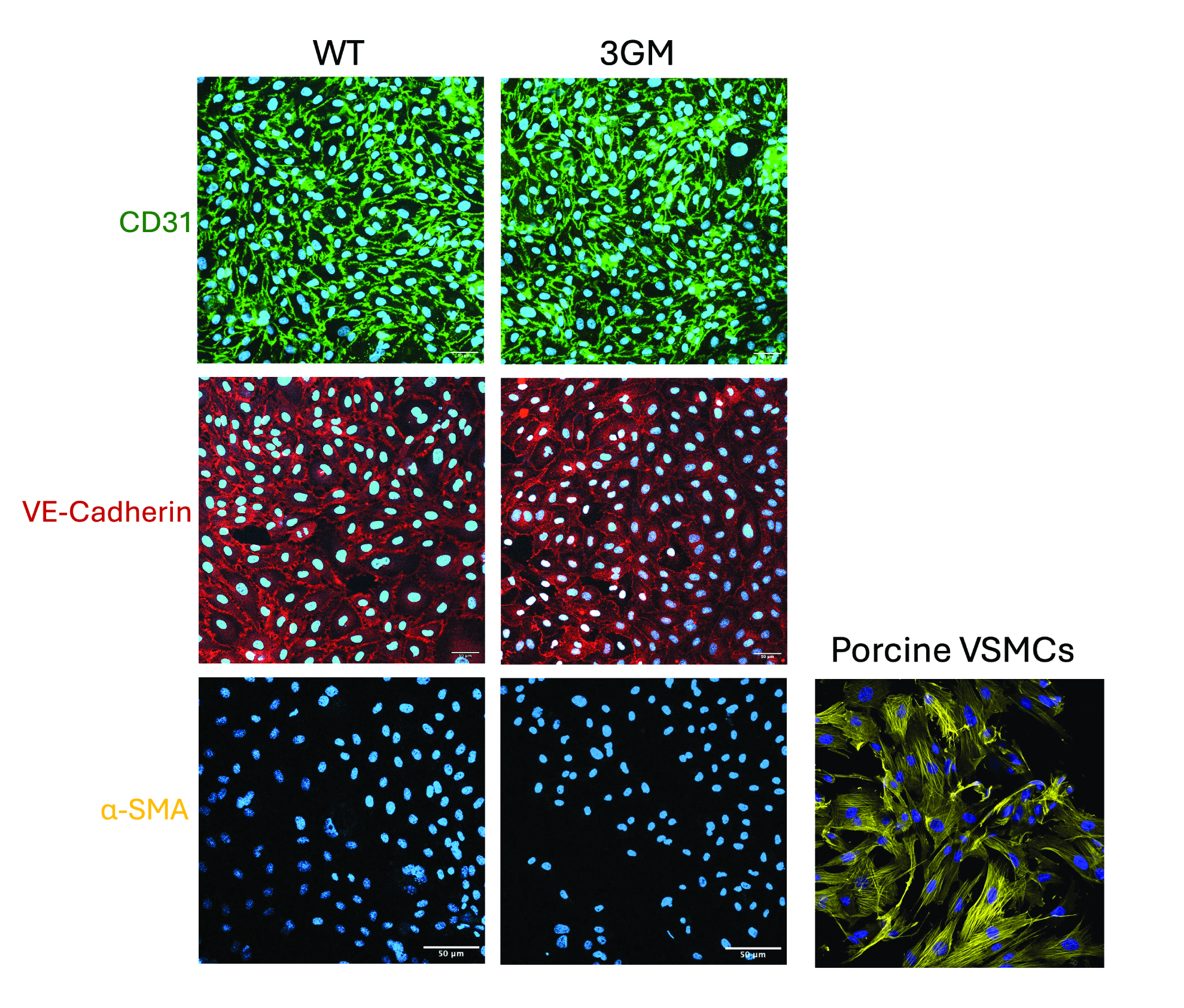

Supplement: Supplementary file 1 — Supporting Information Figure S1: Endothelial phenotype characterization. Representative images of WT and 3GM cells stained for endothelial cell markers CD31 (green), VE‐Cadherin (Red). α‐SMA (yellow) staining confirms the absence of fibroblasts and phenotypic shift, with porcine vascular smooth muscle cells (VSMCs) as a positive control. Scale bar = 50 µm. [file XEN-33-e70149-s001.tif]

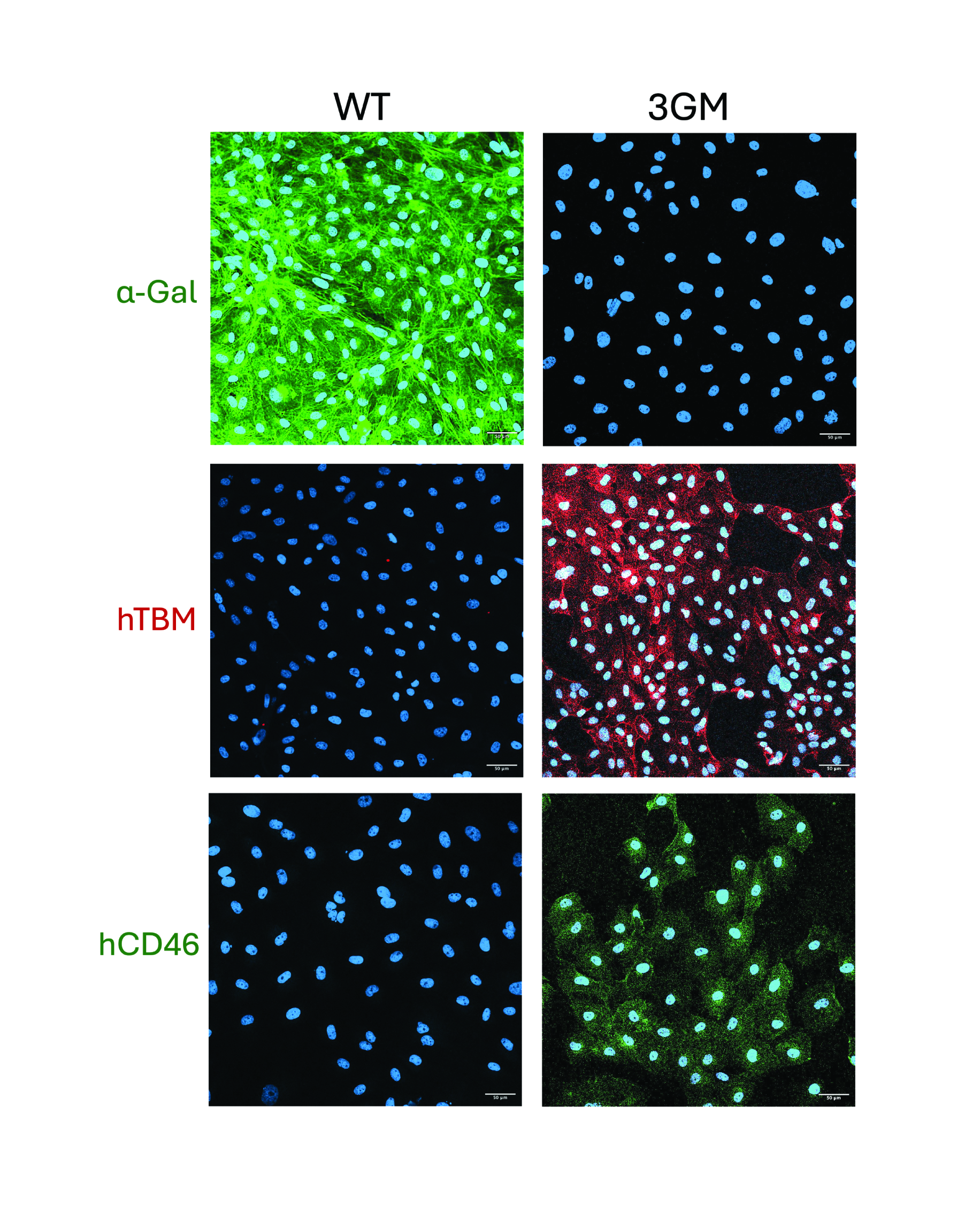

Supplement: Supplementary file 2 — Supporting Information Figure S2: Confirmation of the genetic modification in 3GM porcine endothelial cells. Representative images of WT and 3GM PAECs stained with Griffonia simplicifolia isolectin B to visualize the presence of α‐Gal, and species‐specific antibodies to visualize human thrombomodulin (hTBM) and human CD46. Scale bar = 50 µm. [file XEN-33-e70149-s002.tif]

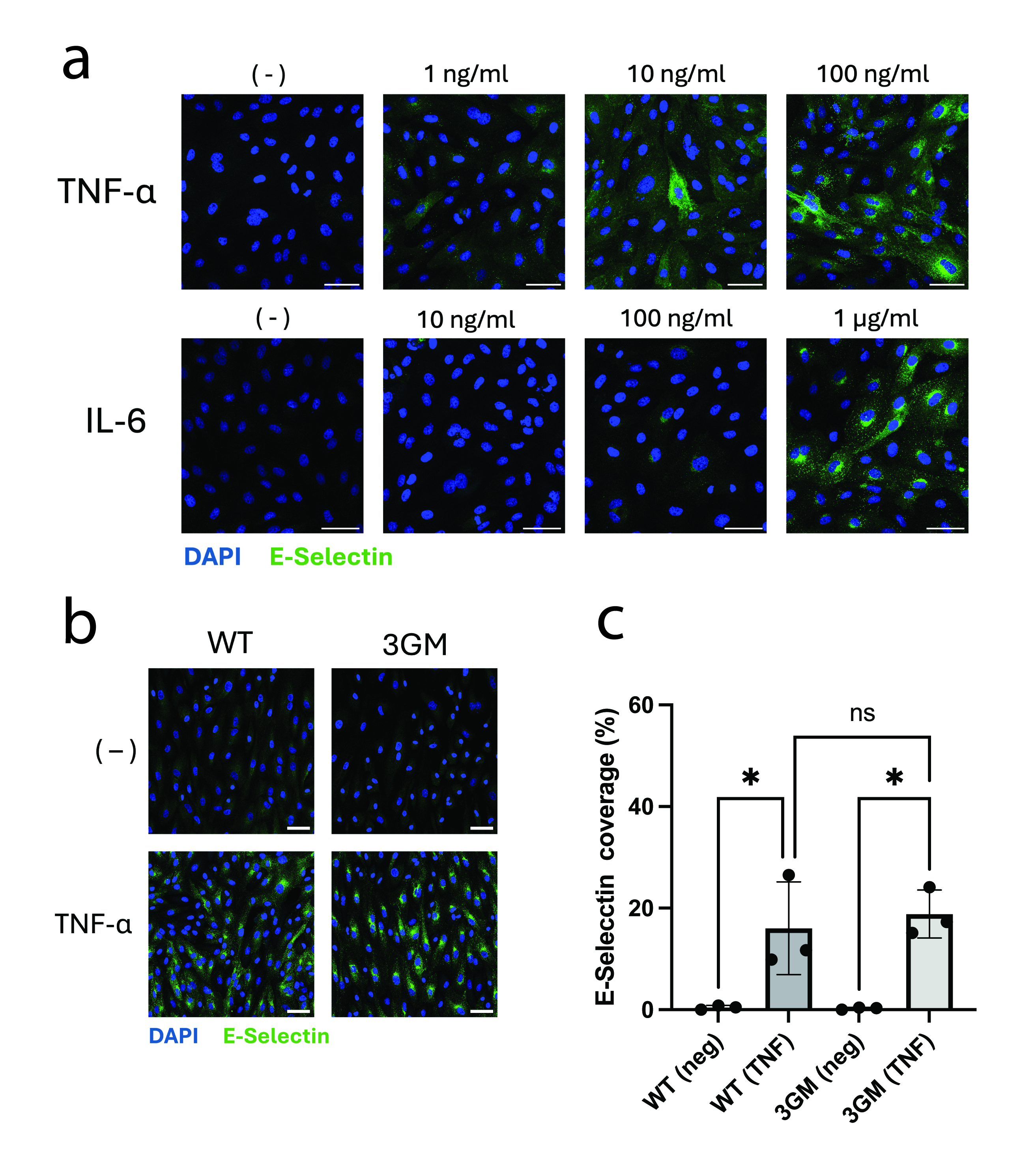

Supplement: Supplementary file 3 — Supporting Information Figure S3: Effects of inflammatory stimuli on endothelial cell (EC) activation. (a) Dose‐dependent effects of TNF‐α (1–100 ng/mL) and IL‐6 (10 ng/mL–1 µg/mL) stimulation, measured by increased E‐selectin expression. (b) E‐selectin expression in WT and 3GM cells following 4 h stimulation with 100 ng/mL TNF‐α. (c) Image‐based quantification of E‐selectin expression from three independent replicates. Asterisks indicate statistically significant differences (p < 0.05). Scale bar = 50 µm. [file XEN-33-e70149-s004.tif]

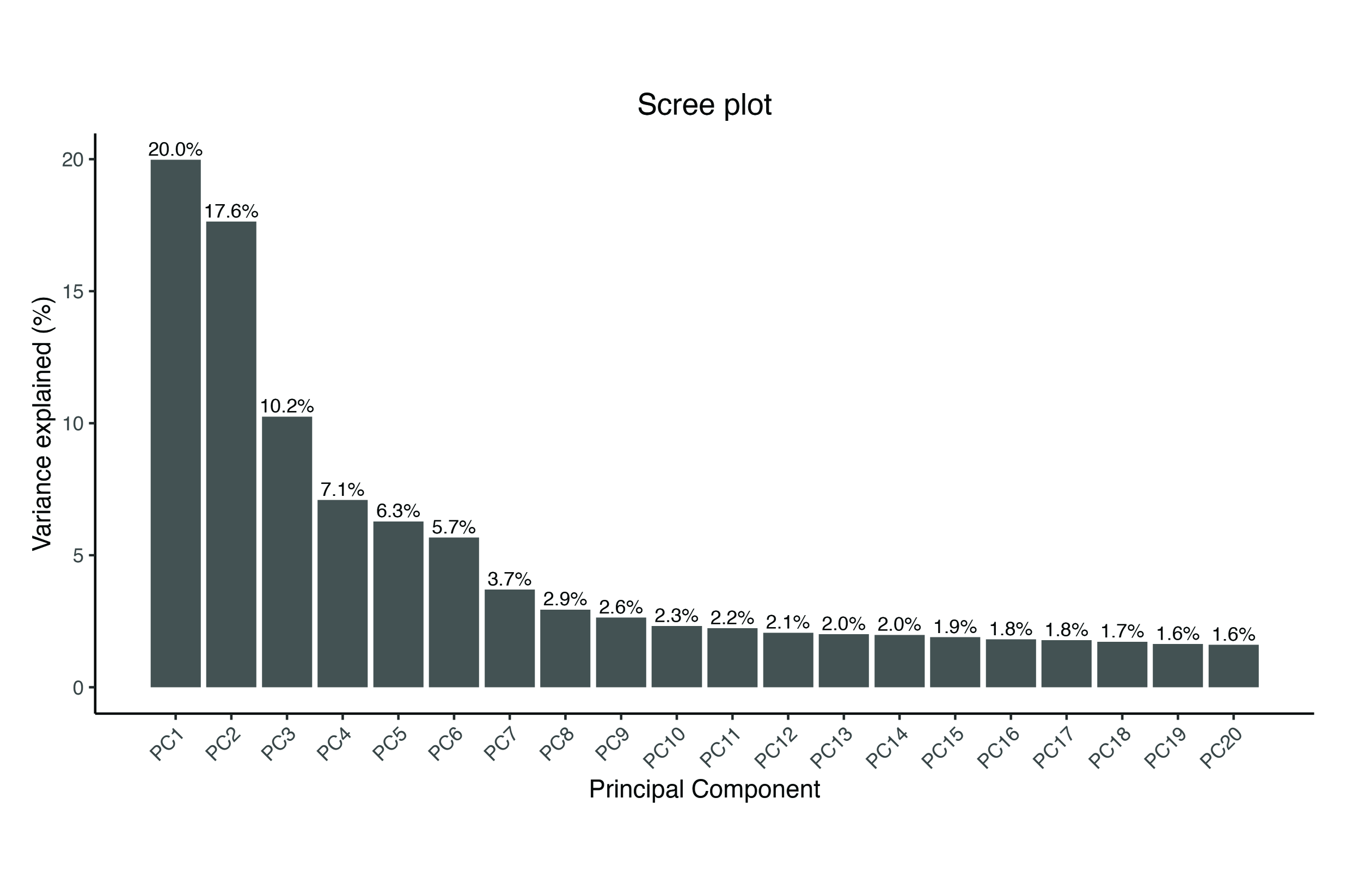

Supplement: Supplementary file 4 — Supporting Information Figure S4: Scree plot from RNA‐seq principal component analysis (PCA) of all cells and treatments, displaying the variance explained by each principal component using 20 gene loadings. [file XEN-33-e70149-s005.tif]

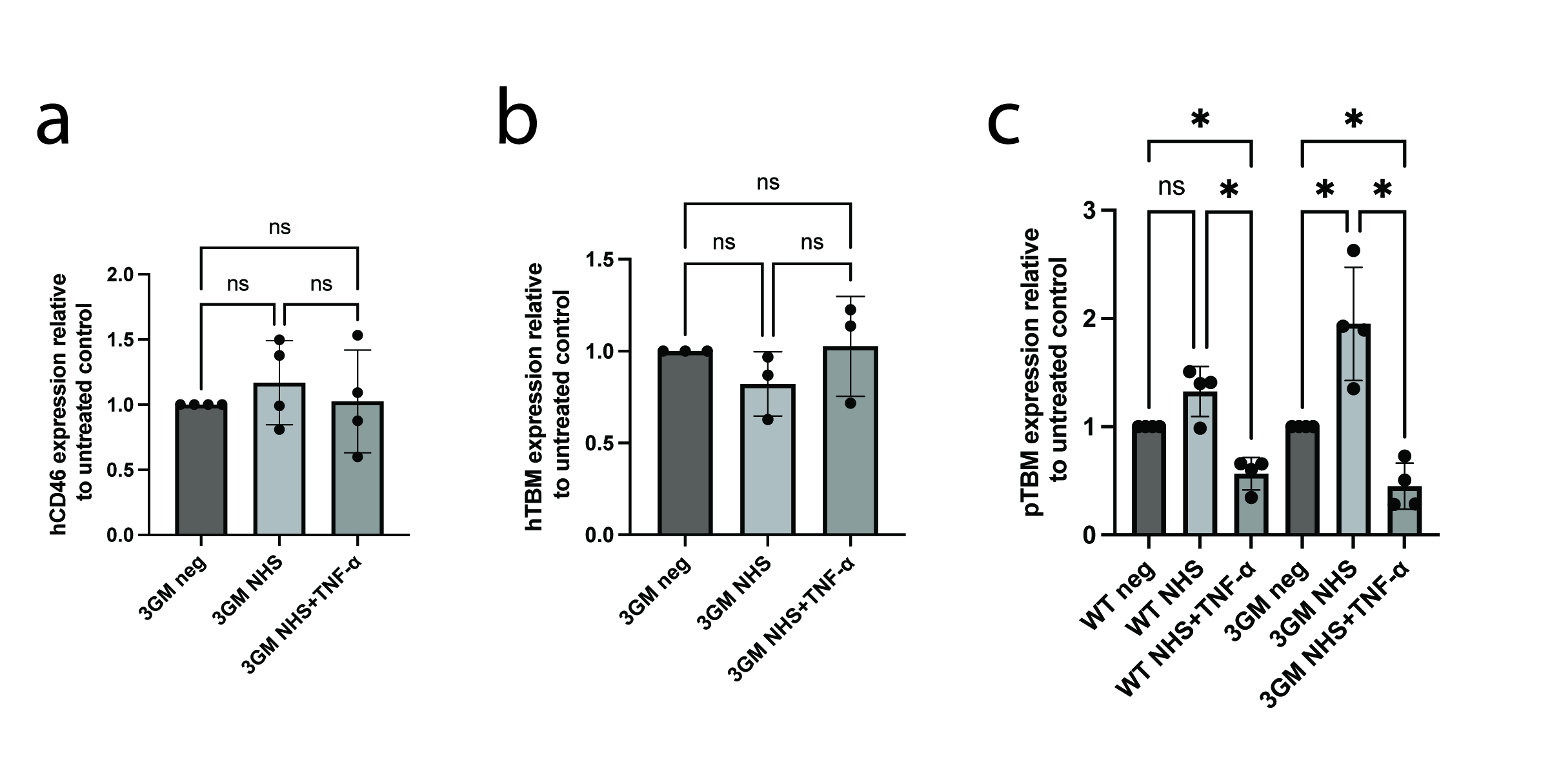

Supplement: Supplementary file 5 — Supporting Information Figure S5: Transgene and endogenous expression of CD46 and thrombomodulin. (a) Human CD46 and (b) human thrombomodulin expression in 3GM PAECs. (c) Porcine thrombomodulin expression in both WT and 3GM PAECs. Expression is displayed as the fold induction of expression in NHS or TNF+NHS‐treated cells compared to untreated cells. Statistical analysis was done using one‐way ANOVA with multiple comparisons. Asterisks indicate statistically significant differences (* p < 0.05). [file XEN-33-e70149-s003.tif]
